# Neural correlation of speech envelope tracking for background noise in normal hearing

**DOI:** 10.3389/fnins.2023.1268591

**Published:** 2023-10-17

**Authors:** HyunJung An, JeeWon Lee, Myung-Whan Suh, Yoonseob Lim

**Affiliations:** ^1^Center for Intelligent and Interactive Robotics, Korea Institute of Science and Technology, Seoul, Republic of Korea; ^2^Department of Electronic and Electrical Engineering, Ewha Womans University, Seoul, Republic of Korea; ^3^Department of Otorhinolaryngology-Head and Neck Surgery, Seoul National University Hospital, Seoul, Republic of Korea; ^4^Department of HY-KIST Bio-convergence, Hanyang University, Seoul, Republic of Korea

**Keywords:** background noise type, informational masking, energetic masking, speech understanding in noise, electroencephalography, neural decoding

## Abstract

Everyday speech communication often occurs in environments with background noise, and the impact of noise on speech recognition can vary depending on factors such as noise type, noise intensity, and the listener’s hearing ability. However, the extent to which neural mechanisms in speech understanding are influenced by different types and levels of noise remains unknown. This study aims to investigate whether individuals exhibit distinct neural responses and attention strategies depending on noise conditions. We recorded electroencephalography (EEG) data from 20 participants with normal hearing (13 males) and evaluated both neural tracking of speech envelopes and behavioral performance in speech understanding in the presence of varying types of background noise. Participants engaged in an EEG experiment consisting of two separate sessions. The first session involved listening to a 12-min story presented binaurally without any background noise. In the second session, speech understanding scores were measured using matrix sentences presented under speech-shaped noise (SSN) and Story noise background noise conditions at noise levels corresponding to sentence recognitions score (SRS). We observed differences in neural envelope correlation depending on noise type but not on its level. Interestingly, the impact of noise type on the variation in envelope tracking was more significant among participants with higher speech perception scores, while those with lower scores exhibited similarities in envelope correlation regardless of the noise condition. The findings suggest that even individuals with normal hearing could adopt different strategies to understand speech in challenging listening environments, depending on the type of noise.

## 1. Introduction

In everyday communication, we encounter a wide range of noise sources that can impede communication in various situations. Individuals with hearing impairments commonly express greater difficulties in understanding speech amid background noise, especially in complex mixtures of spoken content (Roberts and Allen, 2016). Despite considerable research, the precise fundamental mechanisms of the “cocktail party effect,” which denotes the ability to selectively attend to and understand speech in noisy environments.

To quantify speech understanding in noisy environments, evaluations of speech-in-noise perception are essential. Behavioral tests commonly employ two different types of test approach to measure the ability to understand speech in noise. One approach is the fixed-level presentation procedure that maintains a constant intensity of noise level and speech. In general, the final score is calculated based on the percentage of correctly identified sentences, as observed in assessments of speech perception in noise test (SPIN) and connected speech test (CST) (Hutcherson et al., 1979; Cox et al., 1987). However, this approach has limitations, as it does not consider individual thresholds of signal-to-noise ratio (SNR) level and requires multiple sentence test lists to establish the 50% SNR condition. Another approach is an adaptive procedure in which the intensity of the target speech or background noise is adjusted based on individual listener responses. Therefore, the adaptive approach is often employed in the matrix sentence test and the hearing in noise test (HINT) (Nilsson et al., 1994).

Auditory perception studies often place significant emphasis on the task of speech or sound identification within the context of background noise, which can include babble or speech-shaped noise. The overall performance in situations where a speech signal is obscured by background noise is determined by the combined effects of two distinct types of masking (Kidd et al., 1998; Freyman et al., 1999). Energetic masking (EM) (Brungart, 2001) refers to the phenomenon in which the presence of background noise with energy in the same frequency range as the speech signal prevents the clear perception of the speech. However, speech-on-speech masking presents not only the challenge of an overlap in energy frequency bands but also an additional difficulty that cannot be solely attributed to this overlap. Informational masking (IM) (Brungart, 2001) refers to masking in which the signal and masker have similar sounds, making it difficult for the listener to distinguish elements of the target signal from a similar-sounding distractor. IM involves the interference or suppression of one sound by another, where the masking effect goes beyond what would be expected based solely on the physical properties of the sounds involved. This implies that factors other than frequency overlap, such as temporal or spectral cues, cognitive processes, or perceptual interactions, contribute to the masking phenomenon (Durlach et al., 2003; Shinn-Cunningham, 2008). Due to the differing effects of energetic and informational masking, speech perception in noise may be differ depending on the type of background noise. EM, such as speech-shaped noise, primarily contributes to energetic masking by consistently masking speech signals. In contrast, IM, such as single talker noise, offers more opportunities for glimpsing due to temporal variation. However, behavioral measurements (e.g., CST, SPIN, or HINT) fail to specify whether deficits originate from cortical or other mechanisms.

While neural tracking is now the dominant measurement used to explain speech understanding in noise mechanisms, it has been suggested that neural responses reflect slow fluctuations in the amplitude of the incoming speech envelope, indicating a relationship with individual neural responses in central auditory pathways (Shannon et al., 1995; Aiken and Picton, 2008). Neural tracking in continuous speech involves the utilization of neural signals to monitor and interpret speech signals in real time. Previous studies have explored stimulus features, ranging from low-level acoustic characteristics, such as acoustic envelope, onset, spectrogram, and fundamental frequency (Ding and Simon, 2013; Di Liberto et al., 2015; O’Sullivan et al., 2015; Vanthornhout et al., 2018, 2019) to high-level linguistic information such as phonemes sequences, phonetic features, words, or specific word categories like content and function words (Di Liberto et al., 2015; Lesenfants et al., 2019). These attempts suggest that different aspects of speech processing and distinct parts of the auditory pathway mediate neural tracking of various stimulus features.

Several researchers have examined speech processing in noise by analyzing neural responses and their relationship with selective attention to desired sounds in noise scenarios. They suggested that measuring neural envelope tracking can provide insights into the neural mechanisms underlying difficulties in speech perception in noisy environments. For example, Ding and Simon (2013) measured magnetoencephalography (MEG) responses to narrated stories degraded when participants were exposed to varying levels of stationary noise, demonstrating the robustness of neural envelope tracking in the presence of substantial background noise. The study also found a correlation between the degree of neural envelope tracking and perceived speech understanding. Similarly, Etard and Reichenbach (2019) examined neural activity in response to two stories presented in babble noise: one in the participant’s native language and another in an unintelligible foreign language. The results showed an increase in neural envelope tracking with increasing speech understanding only under the condition of the intelligible story, indicating a connection between neural envelope tracking and speech understanding. However, only few studies have compared neural envelope tracking across different types of background noise. The study conducted by Kurthen et al. (2021) examined the effects of energetic and informational masking on neural tracking in healthy older adults. The findings revealed that both IM and EM significantly impede speech-in-noise processing and exert a notable influence on the processing of speech envelopes. However, they had a limited SNR range, which resulted in the inability to account for subjective differences that were not behaviorally matched across the background noise condition.

The current study aims to investigate whether individuals with normal hearing exhibit different neural responses and speech attention strategies under two distinct noise conditions: EM and IM. To achieve this objective, we employed neural envelope tracking using electroencephalography (EEG) and compared the envelope correlations among different groups with varying speech recognition scores. Our findings revealed that neural envelope correlation is influenced by the type of noise rather than its intensity. Notably, the effects of noise type on the difference in envelope tracking were more pronounced when comparing participants with varying speech perception scores.

## 2. Materials and methods

### 2.1. Participants

Twenty volunteers (13 males and 7 females, mean age 26.45 years old) enrolled in the study and provided written informed consent. All participants self-reported having normal hearing and no history of neurological disorders, and all but one were right-handed. All procedures carried out in this study followed the ethical standards of the Declaration of Helsinki and were approved by the Institutional Review Board (IRB) of the Korea Institute of Science and Technology and Seoul National University Hospital (IRB codes: 2017–016 and 1706–137–861, respectively).

### 2.2. Stimuli

#### 2.2.1. Target stimuli

The Flemish matrix sentence test for Korean version (Kim and Lee, 2018) was used in this experiment to assess participants’ speech understanding in a behavioral context. The matrix sentences consisted of five words, each representing a different word category (name, adjective, object, numeral, and verb). For each word category, 10 alternatives were randomly selected to create a set of sentences with similar behavioral speech intelligibility scores. These matrix sentences were designed to sound natural but have grammatically trivial and semantically unpredictable content, thereby minimizing the effect of higher-order language processing on the results. The sentences were equated to have RMS (root mean square) amplitude at a 65 dB SPL.

#### 2.2.2. Maskers

To investigate the impact of different background noise conditions on envelope tracking and speech understanding, three conditions of background noise were added during the presentation of the matrix sentences: The SSN masker was created by extracting the long-term spectral envelope of the matrix sentence and then shaping Gaussian noise with the extracted spectral envelope. The story noise masker was composed of recordings of male speaker, reading separate passages from a narration of a novel (“Marine advantage”) written for children ages 8 to 12 years. The duration of story noise matched each matrix sentence. The story noise was scaled to equal root mean square level (Park et al., 2023).

### 2.3. Experimental procedure

**Figure 1:**
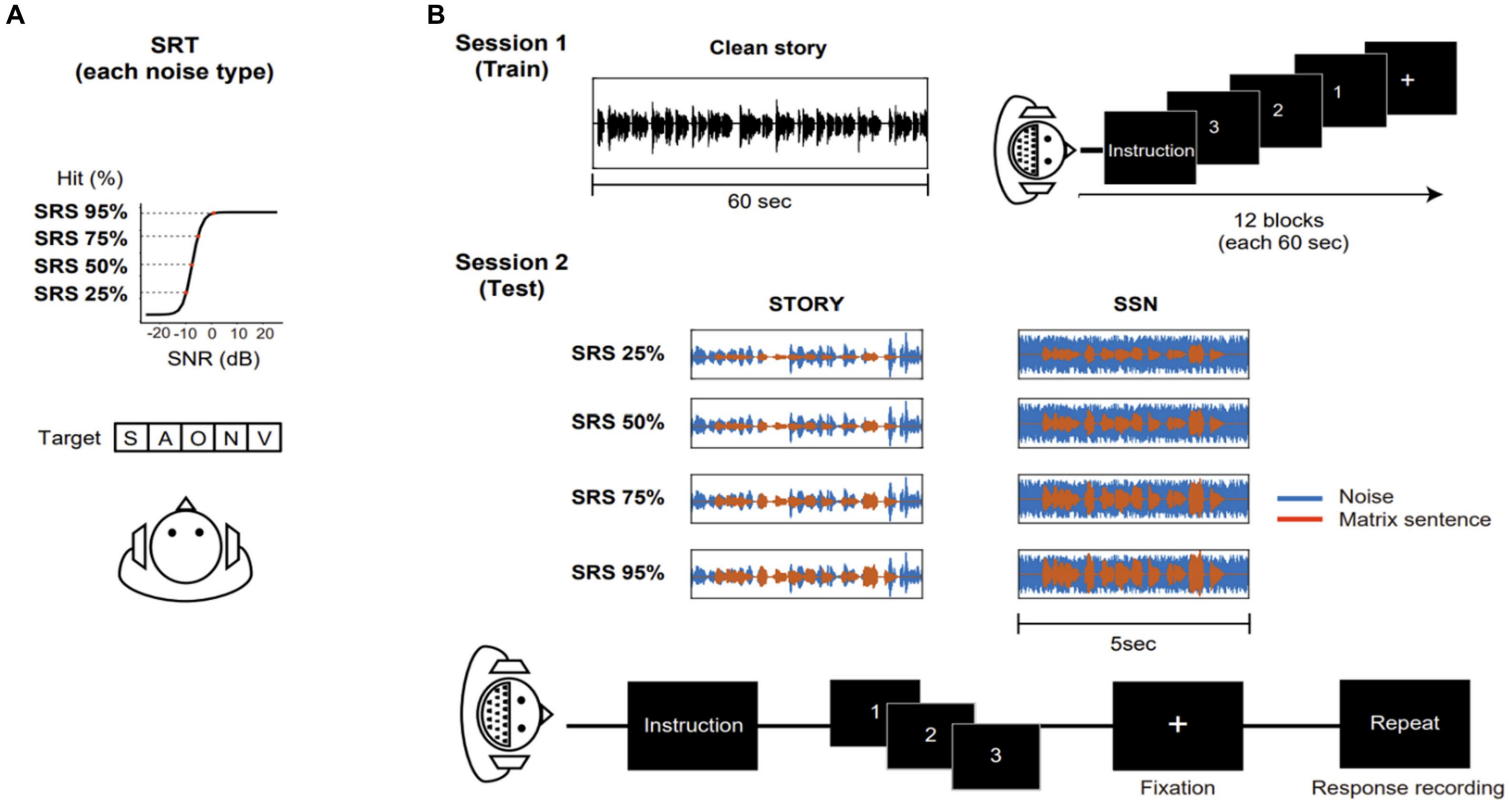
An overview of the speech in noise task conducted during the behavioral and EEG experiment. **(A)** Estimation of speech reception threshold for different noise type. Sentences masked with SSN or story noise were presented and participants were asked to repeat the sentence as they heard it. Speech Reception Score (SRS) were calculated based on the number of correctly repeated word in a sentence. Noise levels corresponding target speech intelligibility levels (SRS: 25, 50, 75, and 95%) were chosen and utilized in the EEG experiment. **(B)** Procedure of EEG experiment. In session 1, to establish a baseline decoder model for speech recognition without any masking, the “Kongjui and Patjui” train story were used (Total duration: 12 min). Each story segment (60 s) was presented while participants maintained visual fixation on a crosshair centered on the screen during each trial. In session 2, two distinct maskers, SSN (speech-shaped noise) and story noise, were utilized at different speech intelligibility levels (25, 50, 75, and 95%) as determined by the Speech Reception Threshold (SRT) test. For each noise condition, a set of 10 sentence stimuli was employed across four noise condition blocks. Participants were instructed to attend to the sentence in noise, and then repeat it within a given time limit (8 s). Corrective feedback was not provided.

**Figure 2:**
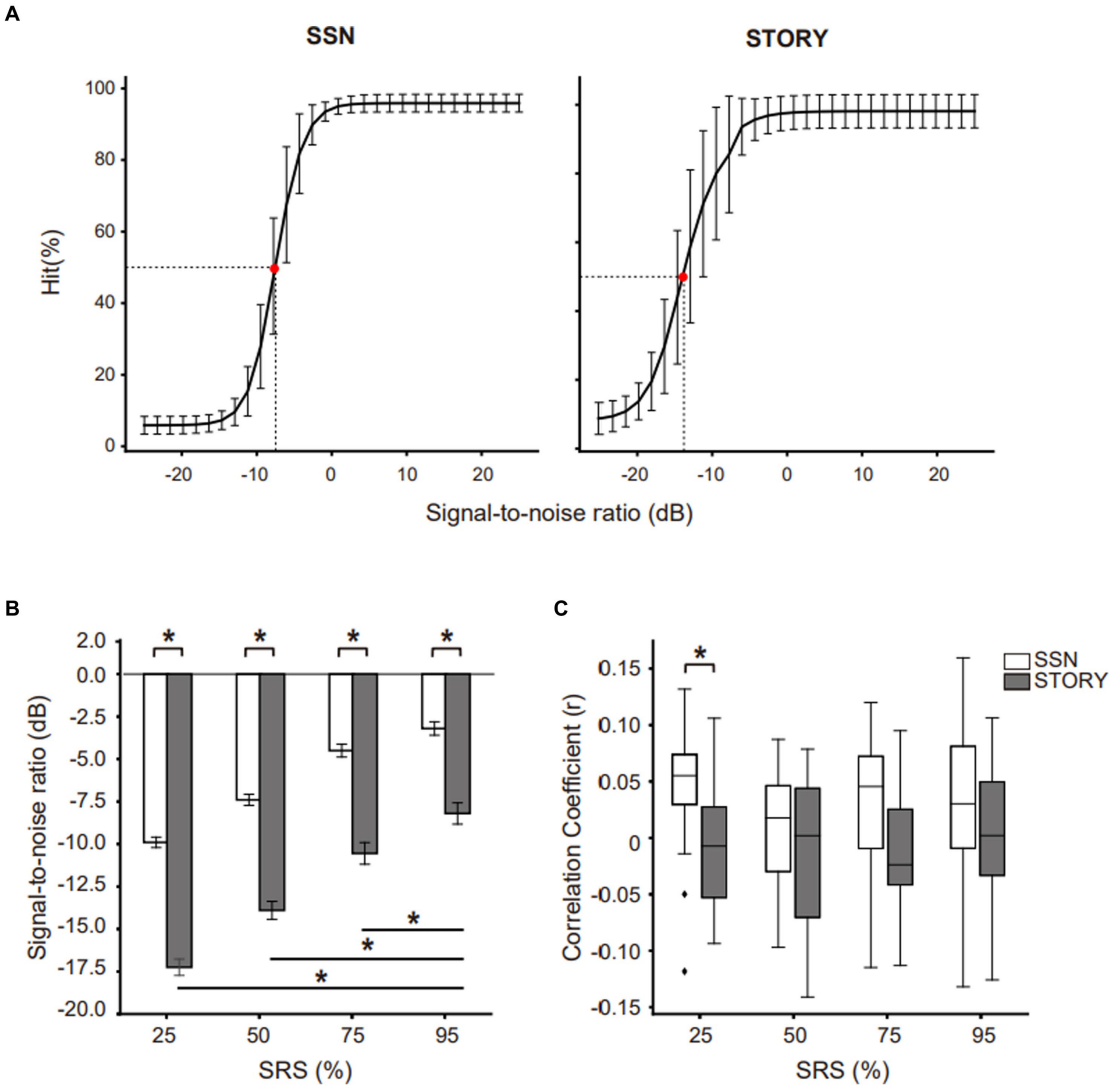
SRT level and envelope tracking results under each noise type throughout the SRS range. **(A)** Each noise type intelligibility function of normal-hearing listeners. The SRS range (25, 50, 75, and 95%) of each noise type in the EEG experiment. Red dot indicates SRT (SRS 50%) for each masker. **(B)** SRT level (dB SNR) for different masker types (white bar: SSN, black bar: Story) throughout the SRS range (25, 50, 75, and 95%). Error bars display the mean ± SEM. **(C)** Neural tracking of the speech envelope is modulated by masker types and the SRS. The center line on each box plot denotes the median neural correlation coefficient, while the edges of the box indicate the SEM. Points outside this range represent outliers. Black bar indicates *p < 0.05.

Prior to the EEG recording, we implemented an adaptive procedure to estimate the speech reception threshold (SRT) for each participant and adjusted the noise level for the corresponding SRT (Figure 1A). During the test, participants were instructed to verbally repeat each sentence as they heard it. The noise level was adjusted based on the number of words correctly repeated in each sentence, ranging from zero to five. If the participant scored below 2, indicating that fewer than two words in a sentence were correctly repeated, the noise level was decreased. However, if the score exceeded 2, indicating that three, four, or five correct words were correctly repeated, the noise level was increased by 1 or 2 dB. The sentence stimuli were presented in the presence of different levels of noise, and the corresponding SRS were determined to achieve 25, 50, 75, and 95% speech intelligibility. Detailed information regarding the SRS for each condition can be found in Figure 2A and Table 1. The range of each noise level was administered for each test condition during the course of the experiment.

**Table 1:** SRT level for each noise type.

Noise type	SRS 25% (dB SNR)	SRS 50% (dB SNR)	SRS 75% (dB SNR)	SRS 95% (dB SNR)
SSN (EM)	−9.9 dB (± 1.37)	−7.4 dB (± 1.46)	−4.5 dB (± 1.7)	−3.2 dB (± 1.76)
STORY (IM)	−17.2 dB (± 2.14)	−13.9 dB (± 2.35)	−10.5 dB (± 2.85)	−8.2 dB (± 2.8)

SRT, speech reception threshold; SRS, sentence recognition score; SSN, speech-shaped noise; EM, energetic masking; IM, informational masking.

The EEG experiment consisted of two separate sessions. In the first session, participants listened to a 12-min children’s story called “Kongjui and Patjui” narrated by a female Korean speaker. This story was presented monophonically without any background noise, providing an optimal condition for creating a linear decoder, as explained in the Data Analysis section. In the second session, the Flemish matrix sentence test was conducted. Participants were instructed to repeat sentences presented under the SSN and Story noise conditions within 8 s. For each noise condition, a set of 10 sentence stimuli was utilized across four SRS blocks, representing SRS 25%, SRS 50%, SRS 75%, and SRS 95%. In total, 40 sentences were presented per condition. The sentences were randomly ordered within each block. The blocks for different conditions were run in a pseudo-random order. To minimize the potential effects of condition order, the block was presented in a different order for each participant, as depicted in Figure 1.

### 2.4. Data analyses

The EEG experiment was conducted in a dedicated soundproof chamber at the Department of Otorhinolaryngology, Seoul National University Hospital. EEG data were acquired using a 64-electrode system, specifically the Neuroscan SynAmps RT with a 64-channel Quik-Cap from Compumedics, Victoria, Australia. The raw EEG signals were recorded at a sampling rate of 1,000 Hz and re-referenced using a common average reference method, excluding the vertical and horizontal electrooculograms. The re-referenced EEG data were bandpass-filtered with a zero-phase Hamming windowed sinc FIR filter with cutoff frequencies of 1 and 8 Hz (v19.1, Delorme and Makeig, 2004). This frequency range has been identified in previous studies (Pasley et al., 2012; Zion Golumbic et al., 2013; Hickok and Poeppel, 2015; O’Sullivan et al., 2015) as relevant for speech envelope processing. To ensure consistent sample lengths for EEG and speech envelope data, both datasets were downsampled to a sampling rate of 64 Hz and normalized through Z-score normalization.

#### 2.4.1. Decoder model

All data analyses were pre-processed using EEGLAB and the mTRF toolbox (v2.0, Crosse et al., 2016) for MATLAB (v9.5.0 R2018b, The MathWorks, Inc., Natick, MA, USA). To quantify neural envelope tracking, we employed the stimulus reconstruction method outlined by Vanthornhout et al. (2018). This approach involved employing a linear decoder to the EEG signals to derive a reconstructed envelope of the stimulus, $\hat{S}\left(t\right)$ . The decoder utilized a spatiotemporal filter that effectively combined EEG signals from multiple channels, as well as their time-shifted counterparts, to reconstruct the envelope optimally. Mathematically, the reconstruction can be expressed as follows:


$$\hat{S}\left(t\right)=\underset{k}{\sum }\underset{\tau }{\sum }w\left(\tau ,k\right)R\left(t-\tau ,k\right)$$

$$w={\left(RR^{T}+\lambda I\right)}^{-1}RS^{T}$$

where $\hat{S}\left(.\right)$ represents the reconstructed envelope, t is the time range, k is the index of recording electrode range, τ is the time lag between the stimulus and the neural response, and R(.) refers to the neural response. The weights of decoder w(.) were determined in a training phase by applying ridge regression with regularization on the inverse autocorrelation matrix. S(.) was obtained by taking the absolute value of the Hilbert transform of the speech signal. A subject-specific decoder was trained for each participant using “Kongjui and Patjui” story, resulting in a matrix size of 64 (EEG channels) x 3,840 (time delay: 500 ms). Following the training phase, the participant-specific decoder was applied to the EEG data of the matrix sentences for each condition, including different SNR levels. Pearson’s correlation coefficient was utilized to measure the correlation between the decoded envelope and the envelope of clean matrix sentence. The time-lag parameter τ was determined within the range of 0–500 ms based on previous research (O’Sullivan et al., 2015). The L2 regularization parameter λ was set to 10^3 based on an iterative search procedure to maximize detection accuracy (Hoerl and Kennard, 1970; Wong et al., 2018).

#### 2.4.2. Statistical analysis

Statistical analysis was performed using R (version 3.3.2) software. The significance level was set at *p* = 0.05 unless otherwise stated. To analyze the envelope tracking under all noise types and sentence recognitions score (ranging from 25 to 95%), we conducted a repeated-measures analysis of variance (ANOVA). The ANOVA included one random factor, which was a participant, and two fixed factors: sentence recognition score (SRS 25, 50, 75, and 95%) and noise type (SSN and Story). F scores and *p*-values from the ANOVA analysis were used to determine the statistical significance of the observed differences. To further explore specific group comparisons and identify significant pairwise differences, we also employed *post hoc* model comparisons using Bonferroni-corrected paired t-tests.

In addition, separate paired *t*-tests were conducted for each sentence recognition score (ranging from 25 to 95%) and noise type to evaluate the influence of different background noise types (IM; Story noise condition versus EM; SSN condition) on participant’s performance. Participants were divided into two groups by their levels of performance (high performance and low performance) based on the median values obtained for each sentence recognition score for the corresponding background story noise conditions. In all our tests, we will compute effect sizes to assess the robustness of our findings. Specifically, an eta square effect size (*η*^2^) of 0.01 will be categorized as a small effect, while 0.06 will indicate a medium effect, and 0.14 will considered as a large effect. For Cohen’s d effect sizes, we will interpret 0.2 as a small effect, 0.5 as a medium effect, and 0.8 as a large effect, respectively (Lakens, 2013).

## 3. Results

### 3.1. Behavioral evaluation of SRT

In this study, prior to EEG recording, the subject-specific SNR were determined for each type of noise (Table 1). To investigate the impact of different types of background noise (SSN and Story) throughout the range (from 25 to 95%, in 25% steps) on the SRT level (dB SNR) among participants, we conducted a repeated measures ANOVA analysis with noise type (2) and SRS range (4) as factors. Figure 2B indicates a significant main effect of SRS range [*F*_(3,57)_ = 407.7476, *p* < 0.05], suggesting that varying SRS had a significant influence on the SRT level among participants. The effect size calculated as eta squared (*η*^2^), was 0.15, indicating a large effect. Post-hoc comparisons demonstrated a significant difference between the SRT levels of SRS 25%, SRS 50%, SRS 75%, and SRS 95%. In addition, type of noise was a significant factor affecting the SRT level [*F*_(1,38)_ = 89.8105, *p* < 0.05], the effect size being relatively large (*η*^2^ = 0.14). The story noise masker (−12.47 ± 4.26) resulted in a lower SRT level than the SSN (−6.25 ± 3.04) masker on average across the SRS range. Furthermore, the interaction between noise type and noise level was observed (*p* < 0.05), with medium effect size (*η*^2^ = 0.06). These results collectively highlight that the SRT level (dB SNR) can vary depending on the type of noise and that under Story noise, the SRT level (dB SNR) is significantly lower than under the SSN condition.

### 3.2. Neural tracking of speech envelope in noise

We examined the influence of SRS range and noise types on envelope tracking. We first calculated the correlation coefficient between the envelope of reconstructed speech and that of the target matrix sentence. The resulting correlation coefficients for both noise types throughout the SRS range are presented in Figure 2C. In repeated-measures ANOVA, we observed a significant main effect of noise type [*F*_(1,38)_ = 5.611, *p* < 0.05], after correction for multiple comparisons and the effect size being relatively large (*η*^2^ = 0.14). Specifically, SSN (0.0462 ± 0.0592) showed a significantly higher correlation value compared to Story noise (−0.0054 ± 0.058) at SRS 25% conditions. However, we did not observe any significant main effect of SRS range (*p* > 0.05) or any notable interaction between noise type and SRS range (*p* > 0.05).

### 3.3. Envelope tracking of better performing listeners in noise

**Figure 3:**
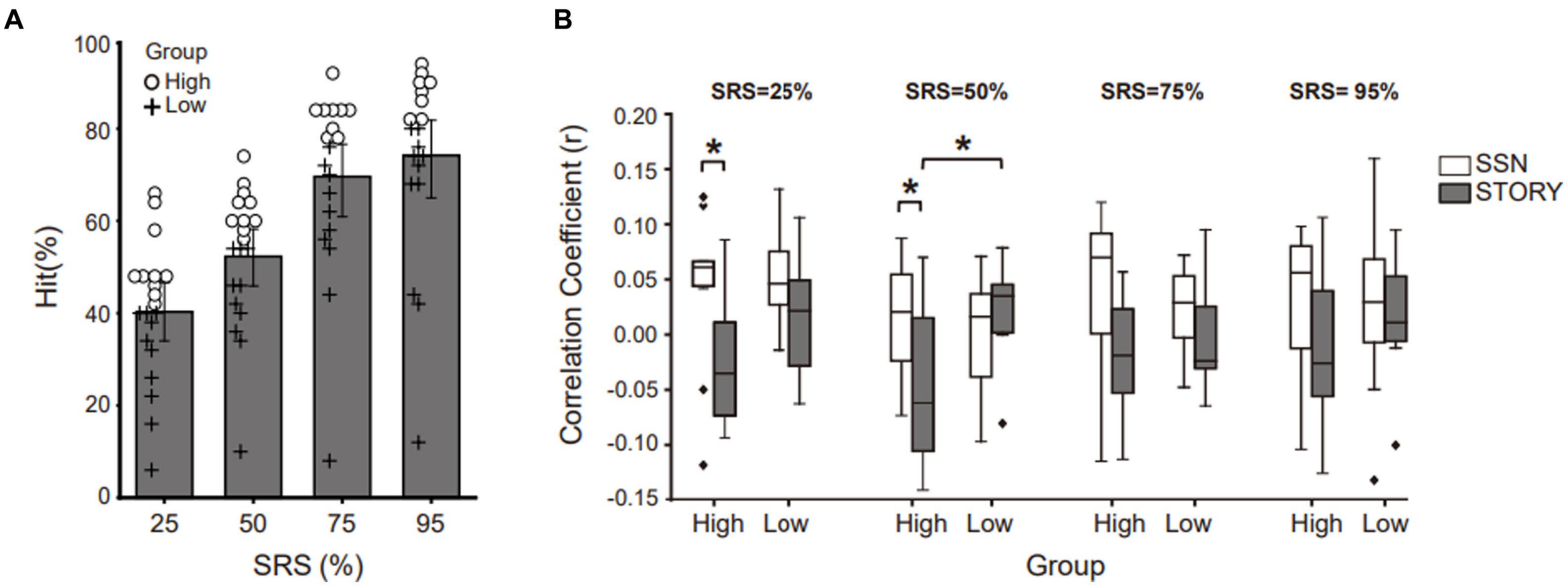
Neural tracking of the speech envelope is modulated by behavioral performance and masker type. **(A)** Behavioral results under the Story noise condition. Participants were divided into two groups based on the median values obtained at each SRS condition for the corresponding background noise conditions. Circles represent the higher-performance group (above median value), while crosses represent the lower-performance group (below median value). **(B)** The neural tracking of speech envelope compared masker type (black bar: Story masker, white bar: SSN masker) within higher- or lower-performance groups across SRS conditions. The center line on each box plot denotes the median neural correlation coefficient, while the edge of the box indicates the SEM. Points outside represent outliers. Black bars indicate *p < 0.05.

The neural tracking experiment revealed an increase in the envelope correlation coefficient under the SSN condition, whereas no such increase was observed under the Story noise condition. These changes were found exclusively in SRS 25% and were not evident in other SRS conditions. To conduct a more comprehensive investigation into the relationship between behavioral performance and envelope tracking with respect to noise type and SRS, we categorized the participants into two distinct groups. This categorization was based on the median values of their behavioral hit scores under the Story noise condition at each SRS (Figure 3A). Subsequently, we conducted paired t-tests to investigate whether significant differences in envelope correlation are present for within-subject factors of noise type and SRS. In Figure 3B, we compared the envelope tracking in SSN and Story noise conditions at SRS of 25, 50, 75, and 95%. Notably, a significant difference was evident within the higher-performance group at SRS 25% (SSN: 0.0420 ± 0.0734, Story: −0.0258 ± 0.0587; t (9) = 2.266, *p* < 0.05) and 50% (SSN: 0.008 ± 0.0561, Story: −0.0539 ± 0.0734; *t*(9) = 2.633, *p* < 0.05, eta squared (*η*^2^) 0.314), the effect size being significant (Cohen’s d = 0.9 and Cohen’s d = 0.7). We also conducted independent t-tests to note any significant between-subject differences that occur due to noise type. We found that the low-performance group showed an overall increase in neural tracking of the speech envelope under the Story noise condition, whereas no clear pattern was observed under the SSN condition. These differences were statistically significant at SRS 50%.

## 4. Discussion

The goal of this study was to investigate the neural tracking of speech envelope in individuals with normal hearing and explore how they perceive speech in noise depending on different noise types, such as SSN and Story noise, as well as throughout an SRS range (25, 50, 75, and 95%). By analyzing the neural responses obtained through EEG, we sought to gain insights into how the auditory system in the brain processes and perceives speech under various challenging listening conditions.

Prior to assessing neural envelope tracking, we evaluated the SRT levels (dB SNR) of different noise types by measuring participants’ ability to repeat a target sentence. The results of the behavioral test revealed that participants exhibited a better (lower) SRT level (dB SNR) in the presence of Story background noise compared to SSN background noise (Figure 2B). According to the traditional perspective, energetic masking (such as SSN) arises from the spectral interference between the target and maskers at the basilar membrane level, whereas informational masking (such as Story noise) occurs at higher processing stages. For instance, cues for segregating speech involve distinctions in the vocal characteristics of the target and masking talkers, such as variations in vocal tract size, fundamental frequency (F0), accent, and speaking style. Furthermore, differences in prosodic features between the target and masking speech, as well as variations in the overall levels of the target and masking signals, also contribute to speech segregation (Darwin and Hukin, 2000; Gustafson et al., 2020). In our study, we used a male speaker to present the Story noise, while the target matrix sentence was presented by a female speaker. By employing distinct cues, such as the gender difference between the speakers, listeners may have successfully distinguished the target sentence from background noise. In addition, the presence of temporal and spectral dips in the Story noise could enhance participants’ ability to effectively detect target cues within the noise, even at higher noise levels compared to SSN. The low SRT levels (dB SNR) observed under the Story background are therefore in line with interpretation that attenuates informational masking (Brungart, 2001).

We observed differences in neural envelope tracking depending on the noise type but not on the level of noise. Specifically, the Story masker exhibited significantly lower envelope tracking compared to the SSN masker at SRS 25% conditions which is the lowest SRT level (dB SNR) (Figure 2C). This finding contrasts with previous studies that reported lower envelope tracking for speech against less noisy backgrounds compared to speech presented against more severe background noise. For example, Das et al. (2018) found that stories without a masker showed lower neural envelope tracking as compared to stories at −1.1 dB SNR. Similarly, Lesenfants et al. (2019) observed that matrix sentences without a masker showed lower envelope tracking in the theta-band when compared to sentences at −3.5, −1, −0.5, and 2.5 dB SNR. This discrepancy between our results and those of previous studies might be explained by different levels of SRT (dB SNR). While previous studies (Das et al., 2018; Lesenfants et al., 2019) used fixed SNR, our study considered behaviorally matched SRT levels (dB SNR). The differences in the experimental study designs could potentially influence variations in envelope tracking for different types of background noise. Participants may individually perceive greater differences in task difficulty and cognitive load when SNRs are fixed compared to adapted SRT levels (dB SNR). Due to a fixed SNR condition, participants with varying hearing abilities and cognitive capacities may experience the task differently due to the one-size-fits-all nature of the auditory environment. In contrast, when SRT levels are personalized, participants are provided with a listening environment tailored to their unique hearing thresholds and cognitive capabilities, potentially leading to varying perceptions of task difficulty and cognitive load among participants (Zekveld et al., 2011).

In our study, we observed notable differences in participants’ performance under different noise conditions. Specifically, in the story noise condition, even with high-intensity noise, participants seemed to be sufficiently informed to process the matrix sentence, which resulted in reduced attention demands compared to the SSN condition. Several previous studies have shed light on the intricate processes underlying speech perception in noise and the factors affecting listening effort. For instance, Dimitrijevic et al. (2019) demonstrated that an increase in alpha power is associated with heightened listening effort, similar to the patterns observed in neural envelope tracking. Additionally, studies by Zekveld et al. (2018) and Koelewijn et al. (2012, 2014) explored the relationship between background noise type and pupil size. They found that different types of background noise, such as speech babble noise or white noise, can elicit diverse effects on pupil size. Generally, more complex and unpredictable background noise, such as speech babble, leads to greater pupil dilation compared to steady-state or predictable noise sources, such as white noise. These findings provide valuable insights into the mechanisms of speech perception in challenging auditory environments, suggesting that individuals adapt their attentional resources based on the nature of the background noise, which has implications for understanding listening effort and cognitive processing during speech perception in noise. Furthermore, we found that the higher-performance group, which was characterized by word identification accuracy values above the median, exhibited significant differences in envelope correlation coefficients compared to the lower-performing group in the SSN and Story noise conditions at SRS 25 and 50% conditions (Figure 3). Within the higher-performance group, envelope correlation coefficients were significantly lower in the Story noise condition compared to in the SSN condition. We speculate that the higher-performance group in our study may have leveraged acoustical cues (modulated or unmodulated dips) present in the story noise to extract target speech information while disregarding irrelevant sound information. It is possible that specific structural characteristics of the story noise, such as temporal fluctuations in the envelope or residual cues (e.g., temporal cue, talker gender), create more opportunities for the “glimpsing” effect (Cooke, 2006). The glimpsing effect refers to the strategic utilization of acoustic information by listeners during momentary reductions in masker energy, often referred to as “dip listening,” in order to enhance their comprehension of spoken words in challenging auditory conditions. Consequently, the higher-performance group likely expended less listening effort to process speech with informational masking, such as story noise, which could explain their lower envelope tracking coefficients. Notably, recent research by Raghavan et al. (2023) has proposed distinct mechanisms for encoding glimpsed and masked speech, providing neural evidence that supports the glimpsing model of speech perception.

In conclusion, the study aimed to gain a deeper understanding of how individuals with normal hearing process and perceive speech in challenging listening conditions by investigating their responses to different noise types and SRS levels (%) using EEG analysis. Our findings showed differences in neural speech tracking under SSN (energetic masking) and Story noise (informational masking) masking conditions. Furthermore, significant differences in neural speech tracking were observed between the higher-performing and lower-performing groups under both noise conditions.

## Data availability statement

The raw data supporting the conclusions of this article will be made available by the authors, without undue reservation.

## Ethics statement

The studies involving humans were approved by the Institutional Review Board (IRB) of the Korea Institute of Science and Technology and Seoul National University Hospital (IRB codes: 2017–016 and 1706–137–861, respectively). The studies were conducted in accordance with the local legislation and institutional requirements. The participants provided their written informed consent to participate in this study.

## Author contributions

HA: Conceptualization, Data curation, Formal analysis, Methodology, Project administration, Writing – original draft, Writing – review & editing. JL: Data curation, Visualization, Writing – review & editing. M-WS: Resources, Writing – review & editing. YL: Funding acquisition, Supervision, Validation, Writing – review & editing.
